# Mind your hand during the energy crunch: Functional Outcome of Circular Saw Hand Injuries

**DOI:** 10.1186/1752-2897-4-11

**Published:** 2010-09-06

**Authors:** Matthias Frank, Juliane Hecht, Matthias Napp, Joern Lange, Rico Grossjohann, Dirk Stengel, Uli Schmucker, Axel Ekkernkamp, Peter Hinz

**Affiliations:** 1 Department of Trauma and Orthopedic Surgery, Emergency Department, Ernst-Moritz-Arndt-University, Sauerbruchstr., 17475 Greifswald, Germany; 2 Center for Clinical Research, Department of Trauma and Orthopedic Surgery, Unfallkrankenhaus Berlin, Warener Str. 7, 12683 Berlin, Germany

## Abstract

**Background:**

Although injuries due to circular saws are very common all over the world, there is surprisingly little information available about their functional outcomes. As the socioeconomic impact of these injuries is immense and determined by the casualties' disability and impairment, it is the objective of this study to present data on the functional outcome, disability, and impairment of hand injuries due to electric circular saws.

**Methods:**

Patients treated from 1999 through 2007 for circular saw-related hand injuries were contacted and asked for clinical follow-up assessment. The clinical follow-up protocol consisted of a physical examination and an assessment of static muscle power (grip and pinch strength). For assessment of the subjective experience of the patients regarding their injury-related disability and impairment, the DASH follow-up questionnaire was used. The occupational impact of these injuries was measured by number of lost working days. Finally, safety-related behaviour of the patients was investigated.

**Results:**

114 Patients were followed-up on average 52 months after the injury. Average in-house treatment was 8.8 days. Average time lost from work was 14.8 weeks. A significant reduction of static muscle testing parameters compared with the uninjured hand was revealed for grip strength, tip pinch, key pinch, and palmar pinch. Average DASH score was 17.4 (DASH work 15.8, DASH sports/music 17.7). Most patients had more than ten years experience in using these power tools.

**Conclusion:**

The everyday occurrence of circular saw-related hand injuries followed by relatively short periods of in-house treatment might distort the real dimension of the patients' remaining disability and impairment. While the trauma surgeon's view is generally confined to the patients' clinical course, the outcome parameters in this follow-up investigation, with loss of working time as the key factor, confirm that the whole socioeconomic burden is much greater than the direct cost of treatment.

## Background

More than 31,000 non-occupational table saw-related injuries treated annually in U.S. Emergency Departments are estimated by data analysis from the National Electronic Injury Surveillance System (NEISS) of the U.S. Consumer Product Safety Commission (CPSC) [1].

The economic impact of these injuries is reported to be immense. Hoxie et al. reported the mean cost of medical expenses to be $ 22,086, with another $ 8,668 for lost wages, making a total of $ 30,754 mean cost per circular saw related injury [2].

Though injuries due to circular saws are very common all over the world, there is surprisingly little information available about their functional outcomes. Previous studies mainly focus either on subgroups with specific injury patterns due to circular saws or specific treatment procedures (e.g. replantations following amputations) or on medicolegal issues regarding possible cases of insurance fraud [3,4].

While statutory accident insurers make great efforts to reduce occupational injuries due to circular saws, there is an increasing incidence of hazards due to power tools during do-it-yourself or recreational activities [1]. The socioeconomic impact of injuries due to circular saws has to be evaluated by examining of the casualties' disability and impairment. Therefore, it is the objective of this study to provide data on functional outcome, disability, and impairment of hand injuries due to electric circular saws.

## Patients and Methods

Patients treated from 1999 through 2007 at the authors' Department for Trauma and Orthopedic Surgery as a result of unintentional circular saw-related hand injuries were contacted by telephone and letter and asked for a clinical follow-up assessment. Patients' informed consent was obtained.

The follow-up was performed according to our recently reported assessment protocol [5]. The functional outcome was evaluated by a physical examination. Static muscle power was assessed by measuring grip strength, tip pinch, lateral key-pinch and palmar pinch (three-point pinch) using a hydraulic BASELINE hand dynamometer (Smith&Nephew, Inc., Germantown, MD) and a hydraulic pinch gauge (SAEHAN Corporation, Masan, Korea). Grip strength was evaluated using position 2 on the hand dynamometer (second handle position from the inside) [6]. In each subtest, three trials were averaged [7]. As recommended by The American Society of Hand Therapists, the participant was seated in an upright chair with the arm adducted, the elbow flexed to 90° and the forearm in neutral position. The wrist was held in approximately 30-degree angle of extension and neutral radioulnar deviation [8]. The results were correlated with the uninjured side, the reduction of grip or pinch strength was expressed as a percentage of that of the unaffected hand. The differences in grip or pinch strength between the injured and uninjured sides were compared using the two-tailed Student's t test. The level of statistical significance was set at the probability value of less than 5% (p < 0.05). Statistical analysis was performed using SPSS 16.0.1 (SPSS Inc. Chicago, IL).

An 11-points numerical rating scale (NRS) for self-report of pain intensity under neutral conditions and under maximum grip strength was used [9].

The DASH self-report questionnaire (German version 2.0) was used to assess physical function and symptoms (mandatory functional symptoms section containing 30 questions relating to functional activities and symptoms; optional work section and sport/music section containing each 4 questions relating to hand function in specific job-required activities and hand function in sports/music activities) [10]. A 5-point scale was used for responses. The final summative score was converted to a percentage scale with "0" reflecting no disability (good function) and "100" reflecting major disability.

Data on the patients' sustained satisfaction about the outcome (not the treatment), the overall functionality and the aesthetics of the affected hand was evaluated using an 11-point numerical rating scale (NRS) with "0" reflecting total dissatisfaction and "10" reflecting complete satisfaction [5].

The occupational impact of the injuries was assessed by investigating lost working time (interval from injury to return to full-time work). In cases of unemployment or retirement, interval from injury to return to usual activities of daily living was evaluated.

The injury severity of the study population was classified into three categories: Grade I (laceration without affection of neurovascular, osseous, or tendonous structures), Grade II (laceration with affection of neurovascular, osseous, or tendonous structures), and Grade III (subtotal amputation, avulsion, amputation) [11].

Finally, safety-related behaviour of the patients prior to/at the time of accident was assessed (alcohol consumption, experience in using power tools, wearing of personal protective equipment (PPT), removal of safety devices).

## Results

### Study cohort

Of 172 stationary patients treated for circular saw-related hand injuries during the 9-year study period, 114 patients (8 females) could be enrolled in the follow-up examination. The age at the time of injury was on average 49 years (range: 15 to 81 years) while the age at the time of follow-up was on average 53 years (range: 22 to 82 years). The average follow-up period was 52 months, ranging between 7 and 136 months.

In-patient treatment (including days of admission and discharge) was on average 8.8 days (range: 2 to 49 days). Patients returned to full-time work (or to usual activities of daily living in case of unemployment or retirement) on average 14.8 weeks after the injury (range: 1 week to 104 weeks).

The anatomic topography of the injuries of this sample was one of the issues raised in a previous study which focussed on ergonometric aspects of the trauma mechanisms and resulting injury patterns due to circular saws with special emphasis on medicolegal considerations [11]. For a clearer comprehension of the outcome parameters, the topographic results are briefly summarised: All injuries involved one hand. The injured hand was mainly the left non-dominant hand (54 injuries; right dominant hand: 49 injuries, left dominant hand: 9 injuries, right non-dominant hand: 2 injuries). In 50 cases, only one finger was affected, in 61 cases two or more fingers were injured (two fingers: 25 injuries, three fingers: 18 injuries, four fingers: 11 injuries, five fingers: 7 injuries). The mid-hand/forearm was affected in 3 cases. The radial aspect of the hand (thumb/index) was most susceptible to injury.

### Functional Outcome

**Figure 1:**
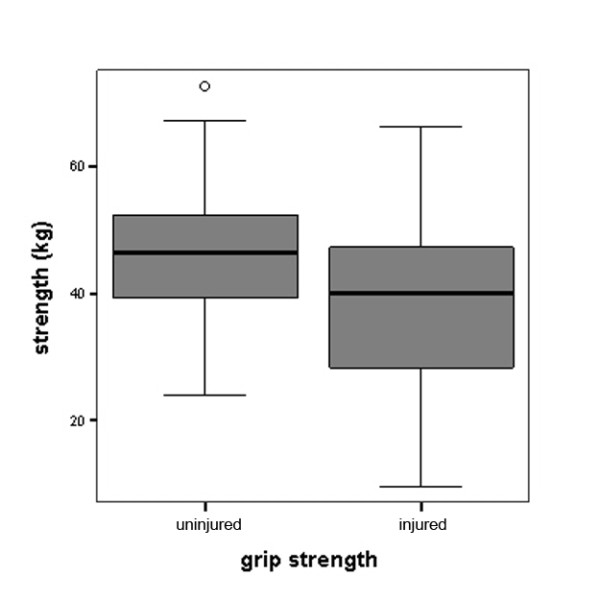
**Grip strength performance of the injured hand compared to the uninjured hand for the whole study population (n = 114)**. Differences were highly statistically significant, for detailed data see Table 1.

**Figure 2:**
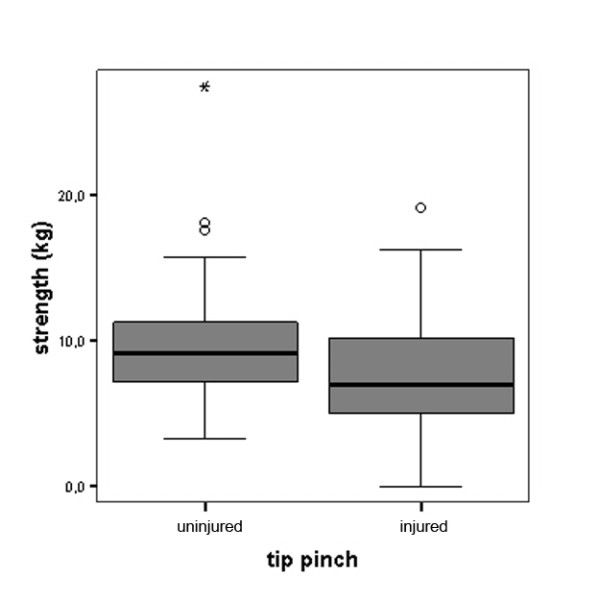
**Tip pinch performance of the injured hand compared to the uninjured hand for the whole study population (n = 114)**. Differences were highly statistically significant, for detailed data see Table 1.

**Figure 3:**
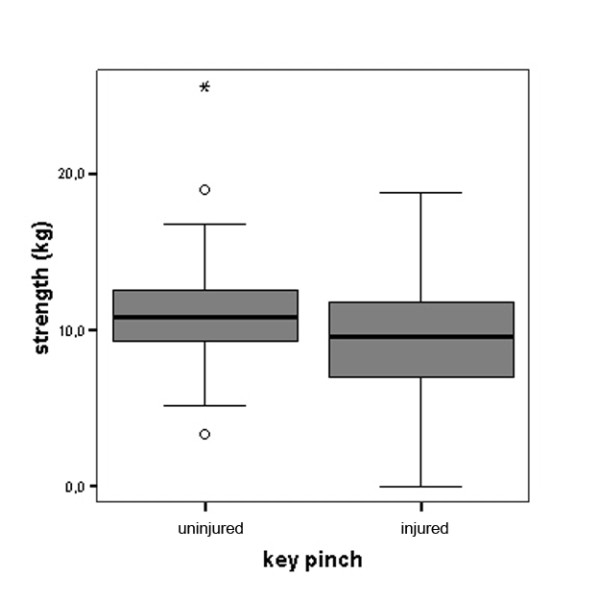
**Key pinch performance of the injured hand compared to the uninjured hand for the whole study population (n = 114)**. Differences were highly statistically significant, for detailed data see Table 1.

**Figure 4:**
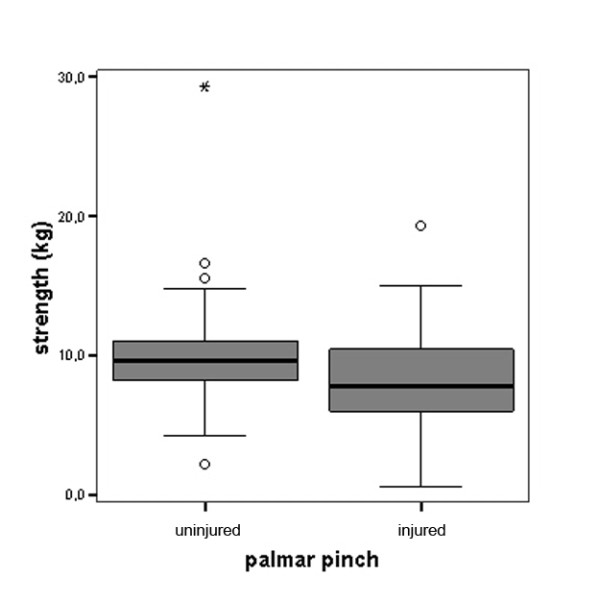
**Palmar pinch (three point pinch) performance of the injured hand compared to the uninjured hand for the whole study population (n = 114)**. Differences were highly statistically significant, for detailed data see Table 1.

For the total study sample, the average difference in grip strength compared to the uninjured hand was -7.73 kg or -16.8% which was highly significant (Figure 1). The average difference in tip pinch was -1.94 kg or -20.6% (Figure 2). Similarly, the average difference in lateral key pinch was -1.74 kg or -15.7% (Figure 3). The average difference in three-point pinch was -1.66 kg or -17.0%, compared to the uninjured hand (Figure 4). All differences were highly statistically significant.

For grade I injuries (laceration without affection of neurovascular, osseous, or tendinous structures), performance of the injured hand compared to the uninjured hand did not significantly differ (p > 0.05) in any subtest, which reflects the complete functional rehabilitation of that injury type.

For grade II injuries (laceration with affection of neurovascular, osseous, or tendinous structures), performance of the injured hand was vastly diminished compared to the uninjured hand (grip strength -6.64 kg or -13.7%, tip pinch -1.6 kg or -17.1%, key pinch -1.29 kg or -11.8%, palmar pinch -0.89 kg or -9.5%) which was highly statistically significant in all subtests.

Moreover, for grade III injuries (subtotal amputation, avulsion, amputation) performance of the injured hand was also vastly impaired in comparison to the uninjured hand (grip strength -9.76 kg or -21.6%, tip pinch -2.39 kg or -25.1%, key pinch -2.27 kg or -20.1%, palmar pinch -2.41 kg or -24.2%) which was also highly statistically significant in all subtests. For detailed data on the static muscle power tests see Table 1.

**Table 1:** Results of the static muscle power tests.

		Grip Strength (kg)		Tip Pinch (kg)		Key Pinch (kg)		Three-Point Pinch (kg)	
		
Injury Severity (number)		Injured Hand	Uninjured Hand	Injured Hand	Uninjured Hand	Injured Hand	Uninjured Hand	Injured Hand	Uninjured Hand
Grade 1 (n = 7)	Mean	44.33	43.20	9.01	9.26	11.06	11.07	11.07	10.37
	SD	9.87	8.53	2.66	2.37	2.15	2.08	3.04	2.19
	Mean Difference (injured-uninjured)	+1.13		-0.24		-0.01		+0.70	
	p	0.435		0.553		0.957		0.216	

Grade 2 (n = 40)	Mean	41.71	48.35	7.74	9.34	9.62	10.91	8.5	9.39
	SD	13.06	11.04	3.59	2.85	3.18	2.72	2.54	2.29
	Mean Difference (injured-uninjured)	-6.64		-1.60		-1.29		-0.89	
	p	< 0.001		< 0.001		< 0.001		0.004	

Grade 3 (n = 67)	Mean	35.38	45.13	7.16	9.54	9.00	11.27	7.56	9.97
	SD	12.87	9.34	3.84	3.61	3.98	3.07	3.52	3.61
	Mean Difference (injured-uninjured)	-9.76		-2.39		-2.27		-2.41	
	p	< 0.001		< 0.001		< 0.001		< 0.001	

Total (n = 114)	Mean	38.29	46.02	7.50	9.43	9.33	11.05	8.12	9.74
	SD	13.09	10.09	3.70	3.25	3.63	2.90	3.27	3.09
	Mean Difference (injured-uninjured)	-7.73		-1.94		-1.74		-1.66	
	p	< 0.001		< 0.001		< 0.001		< 0.001	

Grade 1: Laceration without affection of neurovascular, osseous, or tendonous structures; Grade 2: Laceration with affection of neurovascular, osseous, or tendonous structures, Grade 3: subtotal amputation, avulsion, amputation; SD: Standard Deviation; p: Statistical Significance, Performance was compared using the two-tailed Student's t test

Differences of the grip strength and tip/key/three-point pinch were statistically significant between grade II and grade III injuries (p < 0.001).

Data on the "functional symptoms" section of the DASH questionnaire were obtained from all patients (n = 114). Data on the optional sections "work" and "sports/music" were obtained from 94 (82%) and 63 (55%) patients, respectively. Average DASH score of all patients was 17.4 (range, 0-83.3; SD 18.6), while 14 patients (12%) reported no functional impairment (i.e. DASH score 0). Average DASH work score was 15.8 (range, 0-100; SD 21.8). Average DASH sports/music score was 17.7 (range, 0-100; SD 27.9). No impairment in the work section (i.e. DASH work score 0) was reported in 41 cases (36%), while no impairment in the sports/music section was reported in 36 cases (32%).

With respect to neurosensory complaints hypersensitivity was observed in 46 (40%), cold intolerance in 59 (52%), and paraesthesia in 70 (61%) patients. Constant pain was observed in 10 (9%) patients (average NRS 2.9; range 1-6; SD 1.8), while pain under maximal grip strength was observed in 16 (14%) patients (average NRS 4.1; range 1-8; SD 2.3).

The average sustained satisfaction about the outcome was 7.7 (range, 0-10; SD 2.3), about the overall functionality 6.5 (range, 0-10; SD 2.7), and satisfaction about the aesthetics of the affected hand was 7.7 (range, 0-10; SD 2.5) on the NRS.

For detailed data on the DASH scores, neurosensory complaints, and sustained satisfaction see Table 2.

**Table 2:** Detailed average values of the study data.

Injury Severity	n	**RTW**^**1 **^**(weeks)**	DASH	DASH work	DASH sports/music	**HS**^**2**^ (n)	**CI**^**3**^ (n)	**PA**^**4**^ (n)	Satisfaction Outcome	Satisfaction Function	Satisfaction Aesthetic
Grade I	7	7	3.6	5.4	8.3	0	2	2	9.1	8.1	9.0
Grade II	40	11.7	16.4	19.1	14.8	15	17	23	7.8	6.9	8.2
Grade III	67	16.9	19.3	15.2	23.3	31	40	45	7.6	6.1	7.2
*Total*	*114*	*14.8*	*17.4*	*15.8*	*17.7*	*46*	*59*	*70*	*7.7*	*6.5*	*7.7*

^1 ^RTW: Return to work; ^2 ^HS: Hypersensitivity, ^3 ^CI: Cold intolerance, ^4 ^PA: Paraesthesia

### Safety-related behaviour

While only one patient had no experience at all in using circular saws at the time of the injury (first time user), 86 patients had an experience of more than ten years in using these power tools (the remaining 27 patients stated 1-to-5-year-experience in using circular saws). With regard to frequency of use, 28 patients stated "daily use", 19 patients "several times per week", 8 patients "once a week", and 18 patients "several times per month". Frequency of use was considered as "only sporadically" by 41 patients.

Patients stated the use of personal protective equipment (PPE) in 64 cases. A helmet was used in 6 cases, safety glasses or a face shield in 39 cases, and hearing protection in 17 cases. Working gloves were used in 29 cases. In 13 cases, safety devices such as blade guards, splitter assemblies, or miter gauges, were removed by the operator prior to the injury. In 2 cases, the saw did not have any safety devices. Alcohol consumption prior to the incident was admitted by 8 patients.

After being injured, 26 patients stopped using circular saws, the remaining 88 patients stated a further power tool use.

## Discussion

Working with circular saws is a high-risk activity. While the hand is particularly prone to be injured, severe head and neck trauma [12] and even penetrating thoracic trauma are also reported in literature [13].

After years of a steep decline, rising oil prices are forcing people to use wood stoves all over the world. While sales of modern wood pellet furnace are booming, mainly low-income people, who cannot afford these modern burners, tend to harvest and cut firewood themselves. In rural areas there is also a long tradition of cutting up firewood for personal need.

It is well known from clinical experience and previous studies that injuries to bones and joints as well as injuries to tendons and nerves are significant indicators for being off work and having a prolonged time off work [14]. It is also generally recognised that hand injuries of manual workers result in longer time off work than of other members of the working population [15]. With a blue-collar worker ratio of more than 80% in our cohort, both conditions are applicable to our study sample [11]. The prolonged average time off work of 14.8 weeks in our sample reflects the socioeconomic burden of this type of injury and the extent of indirect cost besides the direct cost (of treatment) [16].

Hand injuries due to power tools (i.e. high pressure injection devices, nail guns) mainly occur in the age groups of the third and fourth decade [17-21]. Circular saw-related casualties are typically older (roughly 50 years on average in this study) due to the high ratio of non-occupational or recreational injuries [2]. As the majority of the casualties of this study stated that they had more than ten years experience in using these tools at the time of the incident, experience seems not to be a protective factor as was previously shown for other power tool-related injuries [5,20].

As described in detail in a previous paper, anatomic location of most injuries was radial-sided (thumb, index or long-finger were affected in 88% of injuries to one finger, in 88% of injuries to two fingers, and in all injuries to three fingers) [11]. Therefore, isometric grip or pinch strength tests evaluating the radial aspect of the hand were employed.

Available information regarding the effect of handedness on isometric grip or pinch strength differences of the right and left hands is inconsistent. While some researchers state that handedness does affect the grip and pinch strength ratio, others report that grip or pinch strength is not or only weakly lateralized [22-24]. To conclude, there is no generally acceptance in literature of the so-called 10% rule (difference in hand strength attributed to hand dominance, with the right dominant hand being expected to be 10% stronger than the left non-dominant hand, whereas the left dominant hand was expected to be as strong as the right non-dominant hand). Due to lack of evidence, this 10% rule was not applied during our between-side comparison. The validity of alternative comparisons between clinically obtained measurements and normative values is limited as they are legitimate only as long as the employed test methods closely resemble those used to obtain the normative values [25].

Prevention is far better than trying to deal with the aftermath of an injury. A recently published study on the trauma mechanisms due to circular power saws revealed the so-called kickback-mechanism as the leading cause of injury [11]. When cutting, ripping or otherwise shaping boards on table-mounted rotary saws, a kickback of the piece occurs when the saw blade becomes hung in the board whereupon the board is rapidly propelled backward into the operator. This causes the stock to bounce out and hit the operator's hand as he tries to stop the stock from flying up and causes his hand to contact the blade. This injury mechanism accounts for roughly 90% of blade contact injuries [1,11]. Various devices and methods have been developed to try to alleviate the problem of stock kickback or to avoid blade contact [1]. However, non-professional wood cutters mainly use non-professional power tools, particularly the smaller and cheaper "consumer models" which regularly lack safety-equipment, such as anti-kickback devices or blade contact avoidance systems.

With regard to personal protective equipment (PPE), hearing protection against noise at high decibel levels and/or exposure periods, safety glasses and face shields against airborne debris or stock kicked-back are strongly recommended. However, there is general agreement that working gloves should never be worn around reciprocating or rotating machine parts, e.g. power saws.

### Limitations of the study

This study has several limitations, the foremost of which is its retrospective nature.

This investigation does not describe the whole number of circular saw-related hand injuries as it encompasses only hospitalized patients and no patients that were discharged after emergency room treatment. However, the number of patients suffering circular saw injuries that were treated and released in our trauma unit is very small compared to the number of hospitalized patients. The outcome data in this study is somewhat limited, as one third of the casualties were lost for clinical follow-up assessment. We still believe, however, that our series is a reasonably representative sample of the patients' personal and the nation's socio-economic burden resulting from this common injury pattern.

## Conclusion

Everyday occurrence of circular saw related hand injuries accompanied by well established treatment algorithms and relatively short periods of in-house treatment might distort the real dimension of the patients' remaining disability and impairment.

While the trauma surgeon's view is generally confined to the patients' clinical course, the outcome parameters in this follow-up investigation (with loss of working time as key factor) confirm that the whole socioeconomic burden is much greater than the direct costs of treatment.

## Competing interests

The authors declare that they have no competing interests.

## Authors' contributions

MF designed the study and drafted the manuscript. JH has been the clinical investigator. MN and JL collected, reviewed and analysed data. RG and DS participated with methods and statistical analysis. US co-drafted the manuscript. AE and PH led project design and steering. All authors reviewed the final draft of the manuscript.
